# DataSHIELD: mitigating disclosure risk in a multi-site federated analysis platform

**DOI:** 10.1093/bioadv/vbaf046

**Published:** 2025-03-10

**Authors:** Demetris Avraam, Rebecca C Wilson, Noemi Aguirre Chan, Soumya Banerjee, Tom R P Bishop, Olly Butters, Tim Cadman, Luise Cederkvist, Liesbeth Duijts, Xavier Escribà Montagut, Hugh Garner, Gonçalo Gonçalves, Juan R González, Sido Haakma, Mette Hartlev, Jan Hasenauer, Manuel Huth, Eleanor Hyde, Vincent W V Jaddoe, Yannick Marcon, Michaela Th Mayrhofer, Fruzsina Molnar-Gabor, Andrei Scott Morgan, Madeleine Murtagh, Marc Nestor, Anne-Marie Nybo Andersen, Simon Parker, Angela Pinot de Moira, Florian Schwarz, Katrine Strandberg-Larsen, Morris A Swertz, Marieke Welten, Stuart Wheater, Paul Burton

**Affiliations:** Department of Public Health, Section of Epidemiology, University of Copenhagen, Copenhagen, DK-1353, Denmark; Department of Public Health, Policy and Systems, University of Liverpool, Liverpool, L69 3GF, United Kingdom; Department of Public Health, Policy and Systems, University of Liverpool, Liverpool, L69 3GF, United Kingdom; BioQuant, Faculty of Law, Heidelberg University, Heidelberg, 69120, Germany; Department of Computer Science and Technology, University of Cambridge, Cambridge, CB3 0FD, United Kingdom; MRC Epidemiology Unit, University of Cambridge, Cambridge, CB2 0QQ, United Kingdom; Department of Public Health, Policy and Systems, University of Liverpool, Liverpool, L69 3GF, United Kingdom; Department of Genetics, University of Groningen and University Medical Center Groningen, Groningen, 9713 AV, The Netherlands; Barcelona Institute for Global Health (ISGlobal), Barcelona, 08003, Spain; Department of Public Health, Section of Epidemiology, University of Copenhagen, Copenhagen, DK-1353, Denmark; Department of Pediatrics, Division of Respiratory Medicine and Allergology, Erasmus MC, University Medical Center Rotterdam, Rotterdam, 3015 GD, The Netherlands; Department of Neonatal and Pediatric Intensive Care, Division of Neonatology, Erasmus MC, University Medical Center Rotterdam, Rotterdam, 3015 GD, The Netherlands; Barcelona Institute for Global Health (ISGlobal), Barcelona, 08003, Spain; National Innovation Centre for Aging, Newcastle University, Newcastle upon Tyne, NE4 5TG, United Kingdom; Human-Centered Computing and Information Science, INESC TEC, Porto, 4200-465, Portugal; Barcelona Institute for Global Health (ISGlobal), Barcelona, 08003, Spain; Centro de Investigación Biomédica en Red en Epidemiología y Salud Pública, Barcelona, 08003, Spain; Department of Genetics, University of Groningen and University Medical Center Groningen, Groningen, 9713 AV, The Netherlands; Centre for Legal Studies in Welfare and Market, Faculty of Law, University of Copenhagen, Copenhagen, DK-2300, Denmark; Life and Medical Sciences (LIMES) Institute and Bonn Center for Mathematical Life Sciences, University of Bonn, Bonn, 53115, Germany; Life and Medical Sciences (LIMES) Institute and Bonn Center for Mathematical Life Sciences, University of Bonn, Bonn, 53115, Germany; Department of Genetics, University of Groningen and University Medical Center Groningen, Groningen, 9713 AV, The Netherlands; Generation R Study Group, Erasmus MC, University Medical Center Rotterdam, Rotterdam, 3015 GD, The Netherlands; Department of Pediatrics, Erasmus MC, University Medical Center Rotterdam, Rotterdam, 3015 GD, The Netherlands; Epigeny, St. Ouen, France; Department of ELSI Services and Research, BBMRI-ERIC, Graz, 8010, Austria; BioQuant, Faculty of Law, Heidelberg University, Heidelberg, 69120, Germany; Elizabeth Garrett Anderson Institute for Women’s Health London, University College London, London, WC1E 6DE, United Kingdom; Obstetric, Perinatal, Paediatric and Life Course Epidemiology Team (OPPaLE), Center for Research in Epidemiology and StatisticS (CRESS), Institut National pour la Santé et la Recherche Médicale (INSERM, French Institute for Health and Medical Research), Institut National de Recherche pour l'Agriculture, l'Alimentation et l'Environnement (INRAe), Paris Cité University, Paris, 75010, France; School of Social and Political Sciences, University of Glasgow, Glasgow, G12 8RT, United Kingdom; BioQuant, Faculty of Law, Heidelberg University, Heidelberg, 69120, Germany; Department of Public Health, Section of Epidemiology, University of Copenhagen, Copenhagen, DK-1353, Denmark; BioQuant, Faculty of Law, Heidelberg University, Heidelberg, 69120, Germany; German Human Genome-phenome Archive, DKFZ, Heidelberg, D-69120, Germany; Department of Public Health, Section of Epidemiology, University of Copenhagen, Copenhagen, DK-1353, Denmark; School of Public Health, Imperial College London, London, W12 0BZ, United Kingdom; Department of Molecular Epidemiology, German Institute of Human Nutrition Potsdam-Rehbruecke, Nuthetal, 14558, Germany; Department of Public Health, Section of Epidemiology, University of Copenhagen, Copenhagen, DK-1353, Denmark; Department of Genetics, University of Groningen and University Medical Center Groningen, Groningen, 9713 AV, The Netherlands; Generation R Study Group, Erasmus MC, University Medical Center Rotterdam, Rotterdam, 3015 GD, The Netherlands; Department of Pediatrics, Erasmus MC, University Medical Center Rotterdam, Rotterdam, 3015 GD, The Netherlands; Arjuna Technologies, Newcastle upon Tyne, NE4 5TG, United Kingdom; Population Health Sciences Institute, Newcastle University, Newcastle, NE2 4AX, United Kingdom

## Abstract

**Motivation:**

The validity of epidemiologic findings can be increased using triangulation, i.e. comparison of findings across contexts, and by having sufficiently large amounts of relevant data to analyse. However, access to data is often constrained by practical considerations and by ethico-legal and data governance restrictions. Gaining access to such data can be time-consuming due to the governance requirements associated with data access requests to institutions in different jurisdictions.

**Results:**

DataSHIELD is a software solution that enables remote analysis without the need for data transfer (federated analysis). DataSHIELD is a scientifically mature, open-source data access and analysis platform aligned with the ‘Five Safes’ framework, the international framework governing safe research access to data. It allows real-time analysis while mitigating disclosure risk through an active multi-layer system of disclosure-preventing mechanisms. This combination of real-time remote statistical analysis, disclosure prevention mechanisms, and federation capabilities makes DataSHIELD a solution for addressing many of the technical and regulatory challenges in performing the large-scale statistical analysis of health and biomedical data. This paper describes the key components that comprise the disclosure protection system of DataSHIELD. These broadly fall into three classes: (i) system protection elements, (ii) analysis protection elements, and (iii) governance protection elements.

**Availability and implementation:**

Information about the DataSHIELD software is available in https://datashield.org/ and https://github.com/datashield.

## 1 Introduction

In the contemporary landscape of data-driven research and technological advancements, data analysis holds a paramount position in scientific inquiry and innovation, which increasingly depend on analysis and interpretation of microdata (Templ 2017). In health and social sciences, the term ‘microdata’ usually refers to data at the level of individuals (Crato and Paruolo 2019). Microdata are invaluable because their information content is often richer than that of aggregated data or that of data from higher-level observational units (e.g. from groups of individuals) (Hand 1992). Microdata allow many scientific questions to be answered while reducing ecological fallacy, and they can increase the statistical power of analyses while facilitating the exploration of heterogeneity.

Nonetheless, the rich information content of microdata is also a serious challenge because microdata are often very sensitive: personally, commercially, or because of extensive intellectual investment in their creation (Livraga 2019). Microdata are associated with higher ‘disclosure risk’—the risk of (accidentally or deliberately) inferring individual-level information that can be used to identify individuals or other primary units of observation (Bethlehem *et al.* 1990, Domingo-Ferrer 2009). The disclosure risk in such microdata cannot be fully eliminated without severely limiting their research potential, and so protective methodologies focus on reducing the likelihood that such a disclosure occurs rather than attempting to guarantee that it cannot.

Sharing of microdata is therefore usually constrained by ethico-legal and social considerations of confidentiality and privacy (Trivellato 2018). In all areas of science, the appropriate governance of microdata may also take account of commercial value and/or intellectual investment. The drive to ensure that all data can be accessed as widely and freely as possible is scientifically desirable and links to the ‘human right to science’, which encompasses both scientific freedom and scientific responsibility (United Nations 1948). However, accessibility must have limits, enabling society, its citizens, and competent professionals to have appropriate oversight and control over the use of data in which individuals or groups have a significant stake (Taylor 2017). This is particularly true in relation to patients and research participants who originally donated the data gathered for furthering scientific research and have not consented to the secondary use of the data, especially if it is outside the scope of altruistic purposes.

The importance of these issues is accentuated when data are to be co-analysed jointly from multiple sources because this increases both the potential routes for disclosure to occur and the legal and technical barriers. Federated analysis tools with integrated disclosure control mechanisms provide technological solutions that can reduce many of the challenges in facilitating the safe access to individual-level data and other classes of microdata (Hallock *et al.* 2021).

### 1.1 The DataSHIELD solution

The DataSHIELD project began in 2009 with the explicit aim to provide an open-source software for addressing privacy and data sharing challenges (Murtagh *et al.* 2012, Wallace *et al.* 2014, Budin-Ljøsne *et al.* 2015). It is designed to be consistent with the FAIR (findable, accessible, interoperable, and reusable) principles (Wilkinson *et al.* 2016) and is continuously updated to be compliant with relevant data protection legislations such as the GDPR (General Data Protection Regulation) and respecting evolving societal perspectives on the use and misuse of personal and professional data by including quality and privacy-preserving (FAIR-HEALTH) (Holub *et al.* 2018) as well as ethical/equitable and reproducible/responsible characterizing elements (FAIRER) (Austin 2020, Murtagh *et al.* 2021). DataSHIELD is also acknowledged in ‘The Goldacre Review’ on the use of health data, commissioned by the UK Secretary of State for Health and Social Care, as an open-source federated health data analysis tool with a large user base (Goldacre and Morley 2022).

The tool is based on a client-server architecture that takes the ‘analysis to the data’, rather than the ‘data to the analyst’ (Gaye *et al.* 2014, Wilson *et al.* 2017). This means the data remain ‘server-side’, on servers managed by the legal data-controller or a responsible data custodian, i.e. behind appropriate network security mechanisms like firewalls and reverse proxies, at the data-holding organization. The role of the ‘client-side’ is to issue analysis commands that are sent to each connected data server and executed separately thereupon. These commands transform data, run analyses, and generate selected summary statistics, which are returned to the client-side. Because the summary statistics are typically low-dimensional, these outputs are not directly disclosive (which is a requirement for DataSHIELD analysis methods). Once the client-side has received the statistics from all sites, those can be combined across all studies, and the pooled results can be displayed alongside study-specific results to the analyst, if requested. Crucially, because the summary statistics are often ‘sufficient statistics’ (Reid 2001) and can include core elements of a full maximum-likelihood analysis—e.g. score vectors and information matrices in generalized linear models—analyses by DataSHIELD are highly efficient. Indeed, in most cases, it is mathematically identical to physically pooling the data from all sources in a central warehouse and subsequently undertaking a standard analysis (Jones *et al.* 2012, 2013).

There are three primary use-cases for DataSHIELD:

1. Analysis/co-analysis of microdata is scientifically desirable but ethico-legal or other governance considerations relating to the sensitivity of the data deem the sharing of at least some of the required data undesirable. For example, legal obligations restrict the sharing of data where individuals may be likely to be re-identified, or where data relate to vulnerable persons or groups who may be particularly harmed should their information be disclosed.
2. A research group wishes to share the information held in its data with others—e.g. to contribute to a large consortium-based analysis—but does not wish to cede control of the governance of those data (including the intellectual property or commercial assets they may represent) by physically handing over the data themselves.
3. A dataset contains data objects that are so large (e.g. images or omics structures such as whole genome DNA sequences) that it is impractical to physically transfer them to the analyst.

DataSHIELD has grown well beyond its initial core components, with more than 20 community-developed packages added to the wider ecosystem (DataSHIELD Community Packages 2024). It is now used extensively in many settings across projects in healthcare, biomedical and the social sciences [e.g. NFDI4Health (NFDI4Health 2018), UnCoVer (Peñalvo *et al.* 2021), TRE-FX (TRE-FX 2023), LifeCycle (Jaddoe *et al.* 2020), EU Child Cohort Network (EU Child Cohort Network 2017), EUCAN-Connect (EUCAN-Connect 2019), ATHLETE (Vrijheid *et al.* 2021), InterConnect (InterConnect 2013), INTIMIC (INTIMIC 2019), RECAP Preterm (Bamber *et al.* 2022), LongITools (LongITools 2020), ORCHESTRA (ORCHESTRA 2020)].

Box 1: *Glossary of the key terms* Microdata: data at the level of individuals

Disclosure risk: the risk of inferring individual-level information from data

Disclosure controls: methods used to reduce the likelihood of the disclosure of personal information

Federated analysis: remote analysis performed on distributed servers

Client-side: a device that issues analysis commands and receives back results co-ordinating parallel analyses across distributed servers

Server-side: a device where the data are stored, and the analysis is conducted

Firewall: a device that monitors and filters incoming and outgoing network traffic

Middleware: the software hosting DataSHIELD on the server-side

R parser: a setting that validates the analytical commands submitted from the client-side to the server-side

In this paper, we describe the interlinked array of systems and measures that are built into DataSHIELD to mitigate disclosure risk. A glossary of the key terms is presented in Box 1. Sections 2–4 describe the key components that jointly comprise the disclosure protection systems (system-level, analysis-level and governance-level elements). Finally, Section 5 discusses how the DataSHIELD disclosure mitigating approach meets existing best practices in data sharing/analysis.

## 2 DataSHIELD system protection elements

**Figure 1.**
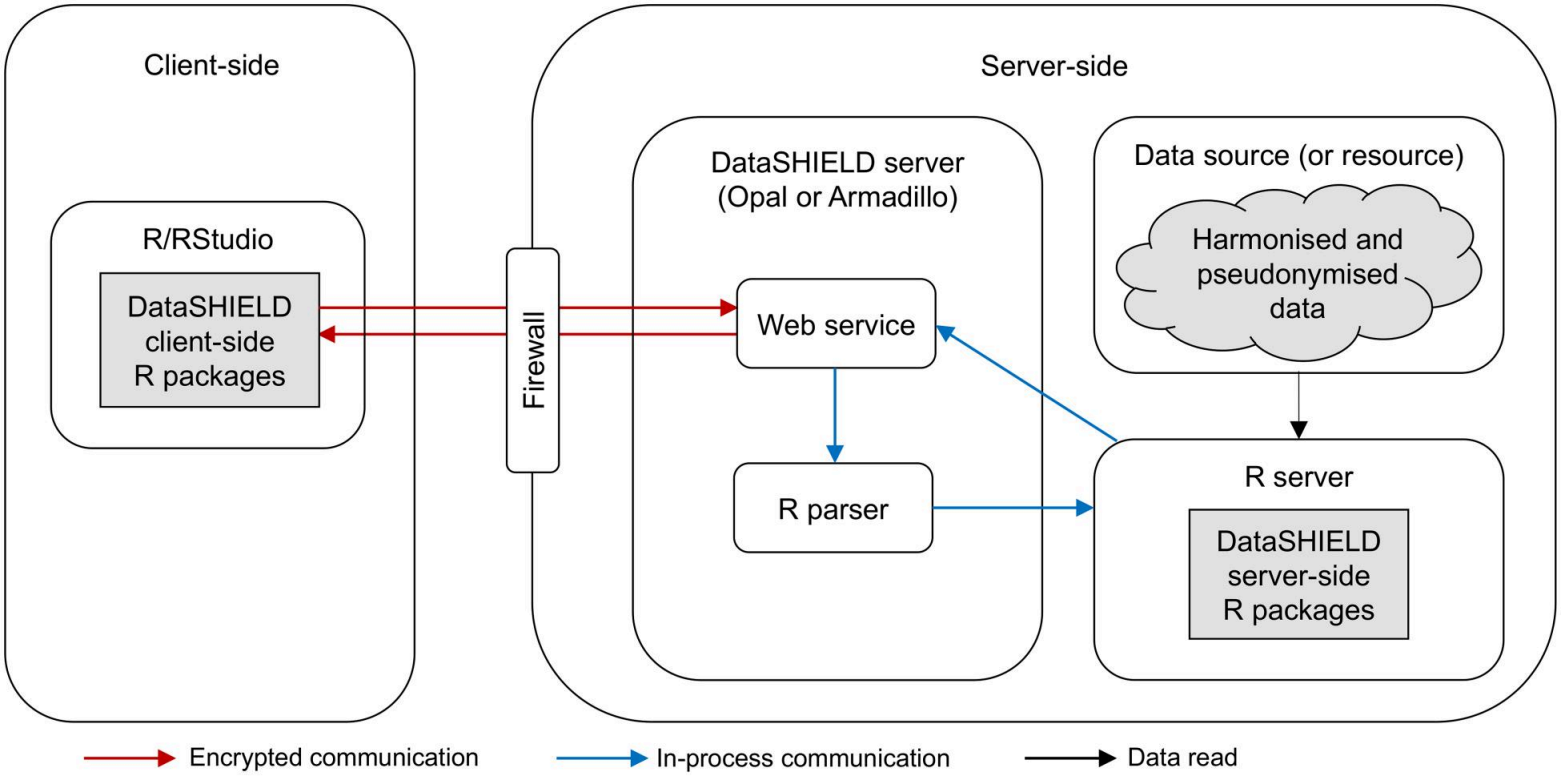
Schematic diagram showing the key DataSHIELD system protection elements.

DataSHIELD utilizes several system-level elements to mitigate the risk of data disclosure that are shown in Fig. 1 and outlined below.

### 2.1 Network security

All traffic is configured to be encrypted. That is, information sent between the client and the server(s) is not inspectable by third parties, even if the traffic passes through their infrastructure. To this end, the client initiates an encrypted https channel to an endpoint on the web service on the server-side. The https connection ports require SSL/TLS certificates and can be configured. Furthermore, a ‘firewall’ protects the endpoint at the web service from other network traffic and blocks unpermitted traffic from entering or leaving the server.

### 2.2 User authentication and authorization

The server-side R environment is set up to only be callable via a middleware hosting DataSHIELD [e.g. Opal (Doiron *et al.* 2017) or Armadillo (Cadman *et al.* 2024)]. This middleware is responsible for authenticating the identity of users. In addition, the middleware also verifies whether an authenticated user is authorized to access a given data set and determines the set of analysis functions they are allowed to use.

### 2.3 R parser

A core aspect of DataSHIELD is that analysts are only able to use special client-side DataSHIELD functions and not native R commands. When applied, these client-side DataSHIELD functions invoke their counterpart server-side functions. The system uses the ‘R parser’, which only allows permitted commands to be called. This prevents commands of known malicious attacks from being executed (i.e. commands that aim to subvert controls and disclose data). For example, the R parser ensures string arguments only contain permitted characters, preventing attempts to pass malicious code to the server.

### 2.4 Data management

The data source should comprise a snapshot, not a live dataset. The data can be either stored in either the Opal or Armadillo database, or kept at their original location, in their original format and be read directly through an R/DataSHIELD server-side session as a resource (Marcon *et al.* 2021). It is hightly recommended that all data sources (particularly snapshots) are pseudonymized. Alongside the described system level protections, studies that deploy DataSHIELD are encouraged to adhere to best IT practices around keeping servers updated and secure, i.e. the operating system is up to date, an anti-malware is installed, etc.

## 3 DataSHIELD analysis protection elements

**Figure 2.**
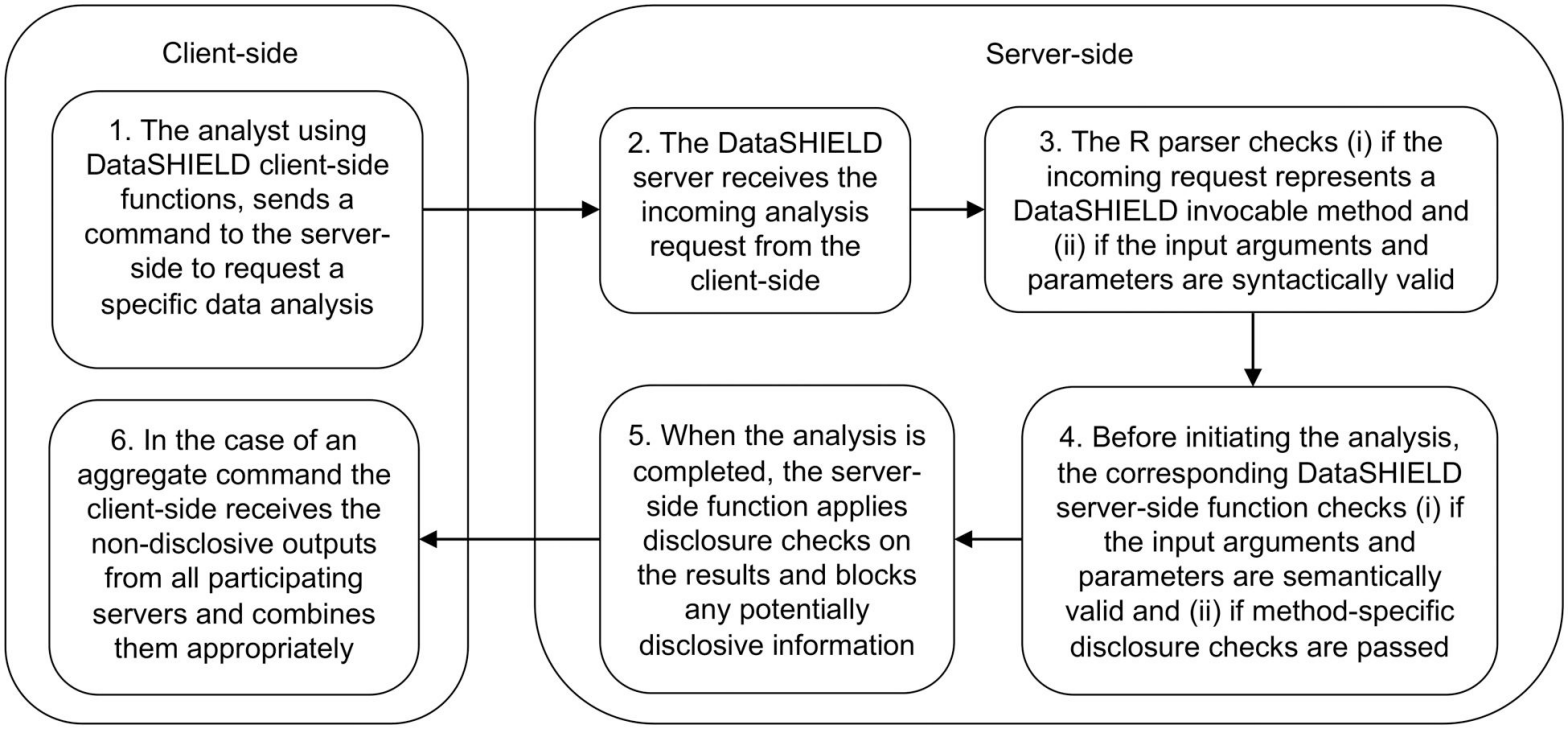
Schematic diagram showing the invocation flow where different disclosure checks and controls are applied during a statistical analysis process in DataSHIELD. Note that this Figure shows a simplified diagram of the invocation flow between the client and a single server. In a multi-site setting, the same flow is applied simultaneously in multiple servers.

Once all system-level checks have been completed, the corresponding server-side function is invoked. Fig. 2 shows the invocation flow where different disclosure checks and controls are applied during a statistical analysis process. In summary, a client-side function sends an analysis request to the servers by calling its corresponding server-side function. The server-side receives the request and, if it is authenticated, it passes the request to the R parser. The R parser checks if the function is invocable and if the included arguments and parameters are syntactically valid. If the request passes the R parser checks, the server-side function processes the incoming analysis request. At this end, the server-side function checks if the input arguments are semantically valid and activates the disclosure controls for the requested analysis (e.g. in a request for fitting a regression model, one disclosure control checks that the number of model parameters to be estimated is lower than a prespecified proportion of the fitted data; see *nfilter.glm* in Section 3.3). Once the analysis is completed, disclosure controls check if the resulting object is non-disclosive and, if yes, the function sends the object as an output to the client-side in the case of an aggregate function or saves the object on the server-side in the case of an assign function (see Section 3.1).

### 3.1 Only use assign and aggregate functions

The incoming analysis requests are restricted to ‘assign’ or ‘aggregate’ methods, which makes it easy to categorize the outcome of any interaction between the client and the server.

Assign functions are those that generate and save objects on the server-side without returning any output to the client-side. Only non-disclosive status messages can be returned to the analyst, e.g. indicating whether the object has been created in the expected format in all studies. An example of an assign function is *ds.asNumeric*, which is based on the native R *as.numeric* function and coerces a server-side R object into a numeric class.

Aggregate functions are those that generate objects on the server-side and return those objects to the client-side. The objects returned to the client-side are aggregated statistical results. The aggregate functions are designed to limit disclosive outputs with the following disclosure control mechanisms: (i) checking inputs to make sure that the function behaves as expected and does not accidentally leak data, (ii) removing outputs that could be disclosive (e.g. residuals and predicted values from regression results) and only returning low-dimensional statistical results, (iii) confirming error messages will not accidentally reveal data. An example of an aggregate function is *ds.table*, which is based on the native R *table* function and generates 1-, 2-, and 3-dimensional contingency tables. The tables are returned to the client-side if they pass appropriate disclosure checks, e.g. enough observations are available such that individual-level data are protected by the aggregation—in this case, meaning that individual cell sizes exceed a minimum threshold level.

### 3.2 Implementation restrictions

The implementation of certain functions in DataSHIELD is restricted. Thus, R functions implemented in DataSHIELD may not return the same information as native R to the client-side. For example, there is no equivalent of the native R *print* function, to prohibit display of values of a server-side object on the client-side computer. Or, unlike R, the DataSHIELD functions for regression models do not return vectors of fitted values and residuals as these may disclose information about individual-level data.

### 3.3 Disclosure controls

Active disclosure controls are embedded in the analytic code that runs on the data processing servers. Collectively, the disclosure controls are used in functions with the intention of only allowing non-disclosive summary statistics (outputs that are not directly disclosive) to leave the server. The disclosure control parameters used by the server-side functions of the *dsBase* DataSHIELD package version 6.3 are listed in Table 1. Other DataSHIELD packages include additional disclosure controls appropriate to the type of data being analysed and documented elsewhere [e.g. *dsOmics* (Escriba-Montagut *et al.* 2024) and *dsSurvival* (Banerjee *et al.* 2022)].

**Table 1.** Disclosure control parameters used by *dsBase* (version 6.3.0) functions.

Name	Description
nfilter.tab	Prevents the return of a contingency table if any of its cells represents less than *nfilter.tab* observations. The value of *nfilter.tab* can be set to any non-negative integer. The default value is set to 3.
nfilter.subset	Prevents the creation of a dataset’s subset if the subset has less than *nfilter.subset* rows. The value of *nfilter.subset* can be set to any positive integer. The default value is set to 3.
nfilter.glm	Prevents the fitting of a regression model that has more than *nfilter.glm x N* unknown parameters in a dataset with sample size *N*. The value of *nfilter.glm* can be set to any numeric value in the interval (0,1). The default value is set to 0.33.
nfilter.string, nfilter.stringShort	Blocks the evaluation of a string argument that passes from the client-side to the server-side, if it has a length greater than *nfilter.string* or *nfilter.stringShort* characters. The values of *nfilter.string* and *nfilter.stringShort* can be set to any positive integers. The default values are set to 80 and 20, respectively.
nfilter.levels.density	Prevents the return of the unique levels of a categorical variable if their length is more than *nfilter.levels.density x N* where *N* is the length of the vector of the categorical variable. The value of *nfilter.levels.density* can be set to any numeric value in the interval (0,1). The default value is set to 0.33.
nfilter.levels.max	Prevents the return of the unique levels of a categorical variable if their length is more than *nfilter.levels.max*. The value of *nfilter.levels.max* can be set to any positive integer. The default value is set to 40.
nfilter.kNN	Prevents the creation of plots (e.g. scatterplot) where the values of the variables to be displayed are obfuscated by their replacement with the centroid of their k-nearest neighbours and k is less than *nfilter.kNN.* The value of *nfilter.kNN* can be set to any positive integer. The default value is set to 3.
nfilter.noise	Prevents the creation of plots (e.g. scatterplot) where the values of the variables to be displayed are obfuscated by the addition of random Gaussian noise with zero mean and variance less than *nfilter.noise* of the true variance of each used variable. The value of *nfilter.noise* can be set to any positive numeric value. The default value is set to 0.25.
datashield.privacy ControlLevel	Permits server administrators to select a predefined subset of the standard methods available. There are currently four modes of operation: ‘permissive’: all functions can be used. ‘non-permissive’: blocks the functions BooleDS, cbindDS, cDS, dataFrameDS, dataFrameSortDS, dataFrameSubsetDS1, dataFrameSubsetDS2, dmtC2SDS, levelsDS, rbindDS, rBinomDS, recodeLevelsDS, recodeValuesDS, repDS, reShapeDS, rNormDS, rPoisDS, rUnifDS, seqDS, setSeedDS, subsetByClassDS, subsetDS, vectorDS that can be used for known inference^a^ or subsetting/difference^b^ attacks. ‘avocado’: blocks the functions BooleDS, cbindDS, dataFrameDS, dataFrameSortDS, dataFrameSubsetDS1, dataFrameSubsetDS2, levelsDS, rbindDS, recodeLevelsDS, recodeValuesDS, reShapeDS, subsetByClassDS, subsetDS, vectorDS that can be used for known subsetting/difference attacks. ‘banana’: blocks the functions cDS, dmtC2SDS, rBinomDS, rNormDS, rPoisDS, rUnifDS, seqDS, setSeedDS that can be used for known inference attacks. The default value is set to ‘banana’.

a Inference attack refers to the process of using information retrieved from statistical analysis to infer individual-level data.

b Subsetting/difference attack refers to the process of inferring individual-level data by comparing two objects (e.g. vectors or subsets) that they differ by one element or row.

The values of the disclosure control parameters are specified entirely by the data custodian(s)—the analyst can see but cannot change these values. For example, the value of *nfilter.tab*, which specifies the allowed minimum count in a non-empty cell of a contingency table, is set to 3 by default. However, the data custodian might decide to change this threshold based on their specific data context and the level of protection they aim to achieve. A value of 1 (no limit) may be necessary, particularly if low cell counts are highly probable such as when working with rare diseases. A value of 5 or 10 is also a justifiable choice to replicate the most common threshold rule imposed by data custodians worldwide (Matthews *et al.* 2016, Ritchie 2022).

### 3.4 Data level obfuscation

Other statistical methods have been implemented in specific DataSHIELD functions or packages to obscure individual-level information and thus to reduce the disclosure risk. Data anonymization is one example of such statistical methods and is used in the graphical functions of DataSHIELD to allow the generation of privacy-preserving data visualizations (Avraam *et al.* 2021). Another example is the use of data synthesis techniques developed in functions of the *dsSynthetic* (Banerjee and Bishop 2022) and *dsBoltzmannMachines* (Lenz *et al.* 2021) packages, which enable the generation of synthetic data. The synthetic data hold the statistical properties and patterns observed on the underlying data stored in the server-side but cannot be used to disclose any original individual-level information. The synthetic data can be used by analysts for prototyping DataSHIELD analysis scripts using R on a standalone computer. This makes the design and development of the analysis plan easier because the synthetic data can be viewed while being manipulated. Once prototyped and tested, the code can be modified for use on the real data via the client-server architecture of DataSHIELD.

### 3.5 DataSHIELD log files

Effort expended on disclosure control should balance the real risk of such disclosure and the real costs associated with it (Hotz *et al.* 2022). Disclosure controls should make it difficult for someone to circumvent them without leaving a trace/mark in the *log files*. The DataSHIELD log files include a permanent record of all commands and is only available to the data custodian managing their data server. This allows *post hoc* investigation of disclosure events by comparing what happened in practice to the formal governance agreements drawn up when the data were made available for analysis and, if necessary, identifying where sanctions should be applied.

There is a crucial distinction between one-step and multi-step methodology for disclosure. Disclosure events that occur with a single analysis step are impossible to detect in DataSHIELD log files or in the contents of the evolving server-side databases, as it would not be possible to distinguish such malicious activity from valid analysis. Any function that allows one-step disclosure must therefore be modified with appropriate disclosure controls as soon as it is identified. Multi-step disclosure algorithms (e.g. Huth *et al.* 2023) have to use two or more functions in a particular order, and generally with a particular structure to the output; hence, there are typically a range of mitigating algorithms that could be run on the server-side log files to identify and alert custodians to disclosure risks. Additionally, there is an opportunity to modify and update the analysis methods exploited by a disclosure algorithm. DataSHIELD is therefore ideally placed to build systems that actively mitigate against more sophisticated forms of attack that attempt to evade baseline protections.

## 4 DataSHIELD governance protection elements

Similarly with all other data sharing or data use practices, formal governance agreements form a crucial part of the DataSHIELD implementation framework. Data access agreements should prohibit DataSHIELD users from attempting to identify individual-level data or data subjects. The necessity of good data governance has been emphasized from the very beginning of the DataSHIELD development (Wolfson *et al.* 2010).

Formal agreements (within and between institutions, with employees granted access to data and external service providers) should govern all steps of a data-use pipeline, from data access to data analysis and finally approval of research results prior to publication. When a research proposal is approved by the data access committee responsible for a data set, users are authorized to analyse only the data listed in their approved application. Hence, only accredited users (e.g. *bona fide* researchers) can login to data servers with user-specific credentials or authentication tokens provided by the custodian(s) of each of the involved data sources.

There are two possible routes a user can take in order to access data on a server-side. The first route is through a local computer that has R and DataSHIELD client-side packages installed. The second route is through a virtual central hub, usually consortium-specific, that a user can have access to through a web portal. The central hub has R with DataSHIELD client-side packages installed. The second route requires another set of user-specific credentials to access the central hub and therefore provides an additional layer of data protection. The central hub can be hosted either by one of the partners participating in a multi-site consortium or by a trusted third party.

It is highly recommended that data uploaded on the servers are pseudonymized [based on the GDPR principle of anonymization and pseudonymization (GDPR 2016a)]. Data pseudonymization (Hintze and El Emam 2018) is the process of replacing direct identifiable information, such as full name, real ID, etc., with a pseudonym. In addition, the granularity of variables is often reduced, e.g. the date of birth is represented only by month and year of birth. Pseudonymized data do not allow an individual to be directly identified without the use of additional information. Additional information linking the pseudonym to directly identifiable information is commonly kept separately and subjected to technical and organizational measures to ensure non-attribution to an identified or identifiable individual. The uploaded-to-the-servers’ data should also be harmonized if they are prepared for co-analysis in a multi-site setting (Fortier *et al.* 2010).

Following approval of data use, the data managers at the data hosting sites authorize users to access the variables listed in their approved application. Thus, an analyst’s access is on a need-to-know basis and only to a subset of variables, i.e. user-specific ‘data views’ [based on the GDPR principle of data minimization (GDPR 2016b)]. In addition, user-specific ‘profiles’ can specify a set of packages or functions that a user can use (see e.g. the *datashield.privacyControlLevel* option in Table 1) and also a set of predefined values for the disclosure control parameters.

Moreover, while DataSHIELD provides an automatic mechanism for output checking based on the disclosure controls outlined in Section 3.3, it is recommended that analysis outputs undergo an additional manual human checking before their release. For example, if an analyst aims to publish the analysis outputs in a paper, the draft should get approval from each data owner or respective body (e.g. research ethics committee) or allow for a review period by such stakeholders before submission of the paper to a journal.

## 5 Discussion

**Figure 3.**
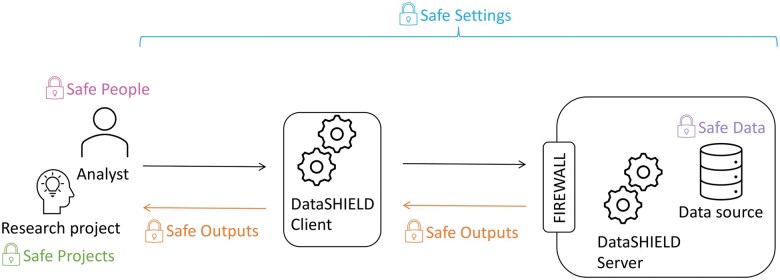
An illustration of the alignment of DataSHIELD with the Five Safes Framework.

Analysis of individual-level data is constrained by ethico-legal requirements and social considerations and by data governance restrictions. DataSHIELD has been developed as a solution to address the above-described barriers of data sharing and access and adopts a multi-layered set of embedded statistical disclosure control methodologies ensuring that appropriate safeguards are in place whilst avoiding the necessity for human checks to be included in the data analysis pipeline. Additionally, the use of disclosure-mitigating policies and processes around the implementation and log-checking that are described in this paper, provide abilities to ensure data disclosure has not occurred. DataSHIELD has been designed to be implemented alongside formal data governance regulations, IT infrastructure best practice and is compatible with the ‘Five Safes’ framework (Ritchie 2017) that has rapidly become a global standard for the safe research access to data (Fig. 3).

The Five Safes framework proposes that data management decisions are considered as responding to five key questions (see Box 2) resulting in ‘safe people’, ‘safe projects’, ‘safe settings’, ‘safe data’, and ‘safe outputs’. Within the context of the Five Safes Framework, DataSHIELD can contribute additional protective elements to existing study mitigations as summarized in Table 2. However, the risk of disclosure can never be completely eliminated, only reduced. If someone with malicious intent has authorization to analyse data, there is the possibility that they may make inferences beyond those intended, via routes that could not have been anticipated ahead of time.

**Table 2.** Examples of study mitigations with their alignment to the Five Safes Framework and how DataSHIELD contributes to these areas.^a^

Five Safes	Study mitigation	DataSHIELD mitigation
Safe People	Formal data access request process Due diligence on prospective users—‘are they bona fide researchers? Have they conducted mandatory training/accreditation to work with data safely?’ Legal contracts, signed terms and conditions of data access and use Sanctions policy **DataSHIELD users are authorized for data access by the study**	The authorization to access DataSHIELD can be delegated, under the principle of subsidiarity to individual studies
Safe projects	Assess the requirement for access to the data—the context of the research project or the data being used Ensuring the data access/use does not contradict any necessary legal requirements, e.g. study consent	
Safe settings	The operation and maintenance of robust computing infrastructure and hardware Following IT security best practice Log of registered users, maintaining/blocking user access Preventative measures for unauthorized access **Deploy DataSHIELD to securely transfer information (analysis commands and outputs) via https** **Each study/consortium provides unique authentication credentials for users to log onto the DataSHIELD client and to connect to each study they are authorized for**	DataSHIELD is a client-server architecture, the user does not connect directly to the study data Analysis of individual level data occurs server-side (at study) Analysis environment server-side can only be called via authenticated users through Opal or Armadillo The server-side R Parser prevents invalid characters or non-approved functions from being run. Users can not directly view the individual-level data User commands logged server-side, only accessible by the study. Can be manually scrutinized e.g. if data misused
Safe data	Data Protection Impact Assessment (risk assessment) Assessing the disclosure risk of the data Pseudonymized data used to lower the disclosure risk	
Safe outputs	Manual checking of analysis outputs for disclosure Legal contracts or terms and conditions making it mandatory for the user to check their own outputs for disclosure before publishing **DataSHIELD disclosure setting thresholds are set and maintained by the study, no one else can alter these**	DataSHIELD server-side functions prevent directly disclosive outputs being returned to the analyst DataSHIELD server-side functions prevent viewing of individual-level data DataSHIELD has disclosure settings based on established statistical disclosure control methods to conduct automated checks for direct disclosure in outputs

a Bold, DataSHIELD mitigation factors that the study controls.

Box 2: *The Five Safes key questions*

- Is this use of the data appropriate? (i.e. is the project safe?)
- Can the users be trusted to use it in an appropriate manner? (i.e. are the users safe?)
- Does the access facility limit unauthorized use? (i.e. are the settings safe?)
- Is there a disclosure risk in the data itself? (i.e. are the data safe?)
- Are the statistical results non-disclosive? (i.e. are the outputs safe?)

### 5.1 Ongoing and future work

The development of a number of novel disclosure controls is planned to continuously mitigate the risks of inferential- and contextual-based disclosures. These new developments will further extend the flexibility of DataSHIELD for the use of datasets that have more stringent data protection requirements and will provide real-time solutions to support and adapt to data governance processes by mitigating the risks. In relation to the analytical functions, new disclosure controls, beyond the conventional Statistical Disclosure Control rules, can be introduced (e.g. advanced methods of differential privacy and homomorphic encryption can be used, as well as Artificial Intelligence). In addition, the DataSHIELD community is now focusing its attention on the development of an automatic monitoring (tracking) of analysis commands that will aim to block any attempts for inferential disclosure (usually multi-step) in real time, as well as, to construct a formal process of auditing DataSHIELD packages provided by developers in the open-source community.

An important feature of the DataSHIELD ecosystem is that it is a free software (What Is Free Software? 2024) licensed under the GNU Public License version 3 (GPLv3) (GNU General Public License 2007). Hence, the wider community is able to review existing code and to submit bug reports and patches, as well as to write and contribute new packages that offer analytic functionalities not currently available. This public availability of source code ensures that the potential discovery of software bugs or statistical disclosure threats is not limited to the developing team. Public disclosure of potential threats allows for faster responses and better solutions than would otherwise be possible from a small, centralized team of developers. It is also unlikely that a single development team would have the resources or knowledge to implement the numerous and diverse statistical methods needed for all possible DataSHIELD use cases. A major challenge for data custodians is the need to trust that all packages have been built with the rigorous considerations around disclosure control in mind. There is currently work ongoing to develop DataSHIELD governance processes around quality assurance and audit processes, as well as for how to respond to potential risks that are identified (DataSHIELD Community Governance Theme 2024).

### 5.2 Conclusion

In conclusion, this paper provides an overview of the non-technical and technical frameworks that must be in place when implementing DataSHIELD alongside respective data governance and operational processes enforced for data privacy protection. This work provides a transparent and actionable framework for addressing privacy in federated data analysis with DataSHIELD and discusses the areas where the implemented mitigation strategies will be further expanded in the future.

## Data Availability

Not applicable.
